# Value-meaning formations in mentally ill patients with a religious worldview

**DOI:** 10.1192/j.eurpsy.2021.1816

**Published:** 2021-08-13

**Authors:** O. Borisova, G. Kopeyko, V. Gusev, E. Gedevani, T. Vladimirova

**Affiliations:** Investigation Group Of Specific Psychopathological Forms At Department Of Youth Psychiatry, Federal State Budgetary Scientific Institution «Mental Health Research Center», Moscow, Russian Federation

**Keywords:** mental patients, religious worldview, Value-Meaning Formations, meaningfulness of life

## Abstract

**Introduction:**

It is necessary to consider the religious worldview and spiritual needs of patients with mental illness in the course of psychotherapy and rehabilitation.

**Objectives:**

The explication of value-meaning formations (VMF) in mental patients with a religious worldview.

**Methods:**

G. Kelly’s methodology of personal constructs (Method of Triads, Hinkle’s laddering technique, the assessment repertory grid by F.Fransella&D.Bannister) and statistical analyses were applied.

**Results:**

1. The structure of the value-meaning formations (VMF) of patients with religious worldview was characterized by its integrity, which is prerequisite for coping. 2. Opposite, patients with absence of religious belief had substantial destruction of integrity and plurality of relationships between VMF. 3. The content of the VMF of mental patients with religious worldview and healthy believers had similarities. 4. In the content of VMF meta-values were: 1. active aspiration to God and the realization of own existence; 2. material well-being in the earthly world; 3. “unselfish” ability to get along without causing harm; 4. feeling of inner confidence.

**Conclusions:**

Mental disease affects VMF of believers and unbelievers in different way. In unbelievers, the structure of VMF in the course of disease significantly changes. In believers, the disease does not destroy the basis of VMF and allows to keep safe the key elements. The stability of VMF in the believers may be explained by the meaningfulness of life. The concepts of “health” and “disease” are included in the worldview of believers, in the general context of their spiritual, psychic and physical life.

**Disclosure:**

No significant relationships.

